# Translation, cultural adaptation, and validation of the Integrated Palliative Outcome Scale-renal (IPOS-r) to Czech

**DOI:** 10.1186/s12904-022-01044-w

**Published:** 2022-08-30

**Authors:** Zuzana Křemenová, Karolína Vlčková

**Affiliations:** 1 Third Faculty of Medicine, Charles University in Prague, Prague, Czech Republic; 2 Kralovske Vinohrady University Hospital in Prague, Šrobárova 50, 10034 Prague, Czech Republic; 3 First Faculty of Medicine, Charles University in Prague, Prague, Czech Republic; 4 Center for Palliative Care, Prague, Czech Republic

**Keywords:** IPOS-renal, Outcome measurement, Validity, Reliability, Patient-reported outcome measure, Advanced kidney disease, Symptom assessment

## Abstract

**Background:**

Patients with advanced kidney disease suffer from burdensome symptoms, which should be assessed by valid and reliable patient-reported outcome measures.

This study aimed to provide a translation, cultural adaptation, and validation of the Czech version of the IPOS-r.

**Methods:**

The IPOS-r was translated to Czech and culturally adapted using cognitive interviews. During the validation phase, patients and staff in dialysis centres and outpatient renal clinics completed the IPOS-r. Internal consistency was tested with Cronbach’s alpha, its reliability via intraclass correlation coefficient for total IPOS-r score, and weighted Kappa (for test-retest and interrater reliability of individual items). Convergent validity was tested with Spearman correlation to Kidney Disease Quality of Life Survey-Short Form 1.2 (KDQOL-SF 1.2). We assessed sensitivity to change using a distribution-based approach.

**Results:**

Two sets of translators independently performed forward and backward translations of the IPOS-r. Ten patients and ten health care professionals participated in cognitive pre-testing. The sample size for validation included 88 patients (mean age 66 ± SD13.8; 58% men) who were treated with haemodialysis (70.5%), home haemodialysis (5.5%), peritoneal dialysis (3%), and conservative management (21%). Cronbach’s alpha was 0.72, and the intraclass correlation was 0.84 for test-retest reliability and 0.73 for interrater reliability. The IPOS-r correlated with KDQOL-SF 1.2 had a rho between 0.4–0.8 for most of the IPOS-r items, showing good convergent validity. The IPOS-r measure is feasible and takes 9 minutes to complete. Patients who reported a change in health status after 1 month demonstrated a total IPOS-r score change of eight points in both positive and negative directions.

**Conclusions:**

The process of translation and cultural adaptation of the IPOS-r was successful, and the Czech IPOS-r measure is a valid and reliable tool. The Czech IPOS-r can be used to assess symptoms in patients with advanced chronic kidney disease.

**Trial registration:**

GAUK [82121].

**Supplementary Information:**

The online version contains supplementary material available at 10.1186/s12904-022-01044-w.

## Background

Patients with end-stage renal disease (ESRD) suffer from a high symptom burden, which is comparable to those living with advanced cancer [1, 2]. Persistent physical or psychological symptoms impair functional status, well-being, and health perception and contribute to a lower quality of life (QoL) [3]. Patients with end-stage renal disease can be treated by conservative management, peritoneal dialysis, or haemodialysis – either in form of home haemodialysis or haemodialysis provided in a health-care centre. These patients have many distressing symptoms that should be assessed by a validated measure. Patients treated with dialysis can live longer, but this survival benefit disappears in frail elderly patients with many comorbidities [4], and their functional status and independence in daily living activities significantly decrease after starting dialysis [5].

The recognition of symptoms and problems by health care staff caring for these patients is often inadequate [2, 6]. They focus mainly on physical symptoms and rely on standard consultation, and the recognition of the severity of symptoms is often poor [7, 8]. Using patient-reported outcome measures (PROMS) on a regular basis can improve QoL and outcomes in advanced kidney disease [9, 10]. The optimal PROMS should be short, sensitive to a change in health status, easy to administer, valid, and reliable for the tested population [11, 12]. There are several PROMS available for renal patients, and some of them are used despite limited validation data [11]. According to a national survey conducted in renal clinics in Australia and New Zealand, IPOS-r was the most frequently used measure [9]. The Integrated Palliative Care Outcome Scale-renal (IPOS-r) was developed by the Palliative Care Outcome Scale (POS) team in the United Kingdom as a result of demand from clinicians to merge the IPOS and the POS-renal. The parent measure IPOS has been validated in a population of palliative care patients with both cancer and noncancer diagnoses, so it is not the best measure for use in renal patients [12]. This was the reason for the development of the IPOS-r measure. IPOS-r contains eleven questions. First two questions contain some symptoms specific to advanced renal disease. From question three to question seven there are psychological domains such as anxiety, depression and feeling at peace, and the last four sentences are about information needs, satisfaction with health care, and practical issues. The English renal-specific version of the symptom checklist, the IPOS-r, shows good test-retest reliability, internal consistency, and construct validity in patients with advanced chronic kidney disease and was recommended for symptom assessment [13]. The IPOS-r offers patient- and staff-completed versions assessing the same domains, both with good psychometric properties [13].

The full parent-measure IPOS has already been translated, culturally adapted, and validated in the Czech Republic, but it is not suitable for renal patients, as the measurement tool was tested on palliative patients in hospices and hospitals, 81% of whom had cancer [14]. The use of the IPOS-r on renal patients has not yet been tested in the Czech Republic.

The IPOS-r has thus far been validated in the English version only, [13] with no country-specific validated translations available. The aim of our study was to bridge this gap and provide a translation, cultural adaptation, and validation of the Czech IPOS-r. To assess the convergent validity of the IPOS-r, we used the correlation to the Kidney disease quality of life-short form 1.2 (KDQOL-SF 1.2), which is the only validated measure that is used in the Czech Republic for assessing the symptom burden of patients with renal disease.

## Methods

This was a mixed-method multicentre study conducted in five facilities in the Czech Republic (one outpatient renal clinic and four dialysis centres). The study was approved by the Ethical Committee of Faculty Hospital Kralovske Vinohrady [EK-VP/I1101202] and the Ethical Committee of Fresenius Medical Care [ekfmc_301/20].

When preparing the study design, we followed the COSMIN checklist for evaluating the methodological quality of studies on outcome measurement [15].

### Concept analysis

The first step was a brief literature review of all concepts used in the IPOS-r followed by the translation and cultural adaptation of the measure.

### Translation and cultural adaptation

This phase was based on guidelines for translation and cultural adaptation of the IPOS family instruments, available on the POS web page [16].

These guidelines are based on International Society for Pharmacoeconomics and Outcomes Research (ISPOR) guidelines [17] and are included in the Mapi Research Trust library specialising in Patient-Centred Outcomes.

Forward translation of IPOS-r was made by two translators with Czech as their first language: one was a health care worker, and the other was a professional translator. Their translations were merged by the research team, and the version for cognitive interviews was created. The Czech version of IPOS-r was then translated back into English by two translators with English as their first language, one with and one without a health care background, and both versions were sent to the POS team in the United Kingdom for the final check.

The final corrected version was used afterwards for cognitive interviews. We performed in-depth qualitative interviews to check the views of patients and staff on the outcome measure.

We reviewed ten patients with advanced kidney disease (three were on conservative management and seven were on haemodialysis) and ten members of the health care team (three physicians, six nurses, and one social worker). We used a convenience sample of respondents who were available and willing to participate in the renal clinic and two dialysis centres at two timepoints. Here is a brief guide to the cognitive pre-testing.

**Table:** 

1. Patient/staff completed the IPOS-r.	
2. We asked them how they understood the questions and the answers and how they chose from them.	
3. We assessed how well they understood the measure and compared their assessment with their answers. In the case of misunderstandings, we asked them what was confusing, and then reformulated the wording.	
4. For every item, we asked if was relevant for them.	
5. Ultimately, we asked if the length of the measure was acceptable and if the recall period was optimal.	
6. We asked if there were any questions that caused discomfort.	
7. All the answers and comments on the measure were written down on the table, which was prepared for this purpose.	

Content analysis of the answers and comments was performed, and the final IPOS-r version was created using patients’ and staff’s views on the measure. We used one-to-one interviews in which verbalization was used to access the thoughts and feelings, and to understand the ideas and interpretations, of respondents who are being asked to process information [18]. We used ‘think-aloud’ technique which was used retrospectively (once a measure was completed).

### Validation

The validation phase was conducted in one outpatient clinic (Faculty Hospital Kralovske Vinohrady in Prague) and four dialysis centres across the Czech Republic (BBraun Avitum Ohradni in Prague, Fresenius Medical Care in Melnik, Fresenius Medical Care in Louny and Fresenius Medical Care in Slany). Data were collected by physicians, nurses, and social workers during regular patient encounters, or patients sent the completed measure by post. We included a convenience sample of adult patients with advanced kidney disease (eGFR < 15 ml/min/1.73m^2^) who were treated with haemodialysis, home haemodialysis, peritoneal dialysis, or conservative management. We excluded those who were cognitively impaired, did not have the Czech language as their mother tongue or were too unwell to participate. Patients were asked to participate by the health care professionals who were involved in the patient’s care. Participants completed the Czech IPOS-r independently or with help from their families or health care provider. Doctors, nurses, or social workers completed their version on the same day independently from the patients.

Measurement data were collected at three time points. Different instruments were used at each time point. At the first time point (T1), patients completed the Czech IPOS-r patient version and the Czech KDQOL-SF 1.2, and health care staff independently completed the Czech IPOS-r staff version. At the second time point (T2), patients completed the Czech IPOS-r 3 days after the first questionnaire had been completed. At the third time point (T3), the Czech IPOS-r was completed 1 month after the first questionnaire, and the patients answered an item asking if their situation had changed since their last completed the IPOS-r. The answer options for this external change criterion were “no”, “yes, negative change” or “yes, positive change”. A negative change meant deterioration of the patient’s overall condition, a positive change denoted an improvement in the patient’s overall condition. It was hypothesized that an improvement in the patient’s overall condition would be associated with a lowering in IPOS-r scores between the time points; deterioration in the patient’s overall condition would be associated with an increase in IPOS-r scores. During the third assessment, patients also completed the time needed to complete the IPOS-r.

### Statistical analysis

Demographic data were reported using descriptive statistics. Patients who had any missing values in the IPOS-r were excluded from the analysis. A significant *p* value was set at 5%, and all analyses were conducted using SPSS version 28.01. We tested the item analysis, reliability, and validity of the Czech version of the IPOS-r as follows:

### Item analysis

For every item of the IPOS-r, we computed the mean and standard deviation. We also computed item difficulty via the individual item‘s mean score and converted it to an interval (0;1) using the formula mean-scale min/(scale max-scale min). Correlations with the total score without a particular item were also provided. Item analysis provides information about the variance of scores and is also used for content validity [19]. Exploratory factor analysis was not done due to the small sample size.

### Internal consistency

The internal consistency was determined via Cronbach’s α for the total score of the IPOS-r.

### Reliability

Two types of reliability were computed. Test-retest reliability was determined based on the first and second assessments of the IPOS-r. We computed the level of perfect agreement for each item with quadratic weighted kappa. The test-retest reliability of the IPOS-r total score was assessed with intraclass correlation coefficient correlations (ICCs). ICCs of 0.7 were considered acceptable, but values > 0.8 indicated high test-retest reliability [20]. Interrater reliability was determined for patient and staff ratings at the first time point using weighted kappa, level of agreement for every item, and ICCs for the total score. The level of kappa from 0.41 to 0.60 was considered moderate, 0.61–0.80 as substantial, and 0.81–1 as almost perfect [21, 22].

### Sensitivity to change

We also assessed the sensitivity to change in our sample using a distribution-based approach [23]. We compared mean changes based on the global change rating, which was assessed by patients during the third assessment after 1 month. Patients were divided into three groups: positive change, negative change, and no change according to their own assessment. The comparison was performed only using descriptive statistics, i.e., the mean change in T1 and T3.

### Validity

To assess the convergent validity of the IPOS-r, we used the KDQOL-SF 1.2, which is the only validated measure that is used in the Czech Republic for assessing the symptom burden and concerns of patients with renal disease. We expected a high correlation (r > 0.70) for items related to the physical status of patients who had similar or identical items in KDQOL and a mid-range correlation (0.5–0.7) between items related to psychological and information needs from IPOS and KDQOL. There was a whole team consensus on the selected items using content analysis. If there were no questions assessing the same concept, we chose those assessing the most similar items; however, some concepts in the IPOS-r were missing in KDQOL (constipation, diarrhoea, sore or dry mouth). To assess validity, we used nonparametric Spearman correlations.

## Results

### Sample

From March 2021 to December 2021, we collected data from 100 patients. However, the IPOS-r data of 12 patients were incomplete and excluded from the final analysis. The final sample consisted of 88 patients with advanced chronic renal disease. The mean age in this sample was 66.1 (SD = 13.8), and 58% of the patients were men. They were treated with haemodialysis (70.5%), home haemodialysis (5.5%), peritoneal dialysis (3%), and conservative management (21%).

### Cognitive interviews

A project team member who is a psychologist with experience in cognitive interviews conducted ten qualitative interviews with renal patients and ten interviews with the health care staff in two dialysis centres and one hospital renal unit. We assessed the face and content validity of the IPOS-r. The interviews covered all questions on the measure. We checked for their comprehensibility, appropriateness, and relevance for the interviewees, and if any problematic parts were found, participants were able to reformulate the IPOS-r questions and answers. All the questions and answers of the IPOS-r were acceptable for the interviewees; we only had to add an explanation of restless leg syndrome, as the concept was not completely clear for those patients who had never experienced it. Participants also suggested adding the “cannot answer” option to the psychological domains of the measure. Health care staff were concerned about the question, “Have you felt at peace?” Although three of the ten thought that patients would not understand the question, none of the patients had any difficulty answering the question.

### Item analysis

In Table 1, we present descriptive statistics—percentages of answers for each value, mean and standard deviation of all IPOS-r items. We also presented the difficulty and correlation of each item with the total score. The minimum difficulty was 0.05 for vomiting, and the maximum was 0.48 for anxiety of family/friends. Most of the item-total correlations were higher than 0.3; only for constipation, diarrhoea, practical problems, and time wasted on appointments was there a lower value. The mean total score was M = 21.8 (SD = 11.3, range 0–47).

**Table 1:** Distribution of scores and item analysis (*N* = 88)

IPOS Item	% response for each value						Mean	SD	Item difficulty	Item total correlations
	0	1	2	3	4	Can not answer				
Pain(i2)	47	15	25	12	1	0	1.1	1.2	0.28	0.43
Shortness of breath(i2)	59	22	11	7	1	0	0.7	1	0.18	0.41
Weakness or lack of energy(i2)	22	33	31	14	1	0	1.4	1	0.35	0.71
Nausea(i2)	78	14	5	2	1	0	0.3	0.8	0.08	0.43
Vomiting(i2)	91	5	3	1	0	0	0.2	0.5	0.05	0.32
Poor appetite(i2)	67	23	8	1	1	0	0.5	0.8	0.13	0.42
Constipation(i2)	74	13	8	3	2	0	0.5	0.9	0.13	0.14
Sore or dry mouth(i2)	48	27	15	9	1	0	0.9	1	0.23	0.49
Drowsiness(i2)	38	26	22	14	1	0	1.1	1.1	0.28	0.64
Poor mobility(i2)	37.5	27	16	12.5	7	0	1.2	1.3	0.3	0.66
Itching(i2)	51	31	8	8	2	0	0.8	1	0.2	0.35
Difficulty sleeping(i2)	44	18	24	9	5	0	1.1	1.2	0.23	0.5
Restless leg(i2)	66	17	10	7	0	0	0.6	0.9	0.15	0.46
Changes to skin(i2)	63	19	0	16	2	0	0.6	0.9	0.08	0.3
Diarrhoea(i2)	81	9	9	1	0	0	0.3	0.7	0.08	0.21
Thirst(i2)	35	32	15	8	10	0	1.3	1.3	0.33	0.51
Anxiety(i3)	47	20	25	6	2	0	1	1.1	0.25	0.42
Family/friends’ anxiety(i4)	27	9	34	15	15	0	1.9	1.4	0.48	0.32
Depression(i5)	62.5	20.5	12.5	4.5	0	0	0.6	0.9	0.15	0.56
Felt at peace(i6)	23	37.5	20.5	15	4	0	1.4	1.1	0.35	0.41
Able to share with family/friends(i7)	37.5	12.5	31	11	8	0	1.4	1.3	0.35	0.36
Information(i8)	47	25	9	8	11	0	1.1	1.4	0.28	0.23
Practical problems(i9)	57	14	12	9	8	0	1	1.3	0.25	0.18
Time wasted on appointments(i10)	54.5	0	41	0	4.5	0	1	1.2	0.3	0.27

Item difficulty is measured with the individual item’s mean score and is converted to an interval (0;1) using the formula mean-scale min/(scale max-scale min)

Item total correlation score refers to correlations with the total score without a particular item

*SD* Standard deviation

### Internal consistency

Cronbach’s alpha for the total score of 24 items was 0.72 [24].

### Reliability

Test-retest reliability was computed for all items and for the total score. We present the mean scores at T1 and after 3 days (T2), the level of perfect agreement between these two ratings, and weighted Cohen’s kappa in Table 2. Most of the kappa coefficients (22 of 24) were above 0.4; only for the items vomiting and information needs was the value below 0.4. The mean at the first time point was M = 21.8 (SD = 11.3), and for the second time point, it was M = 20.1 (SD = 12.1). The ICC for the total score was 0.84 (95% CI = 0.76–0.90).

**Table 2:** Test-retest reliability measured by weighted kappa and level of agreement between T1 and T2 (3 days later) (*N* = 88)

Item	T1 mean	T2 mean	Agreement (%)	Weighted kappa
Pain	1.1	1.1	61	0.53
Shortness of breath	0.7	0.8	68	0.67
Weakness or lack of energy	1.4	1.4	61	0.64
Nausea	0.3	0.2	82	0.46
Vomiting	0.2	0.1	87	0.33
Poor appetite	0.5	0.4	76	0.54
Constipation	0.5	0.4	82	0.67
Sore or dry mouth	0.9	0.7	65	0.58
Drowsiness	1.1	1.1	55	0.55
Poor mobility	1.2	1.3	65	0.69
Itching	0.8	0.8	76	0.75
Difficulty sleeping	1.1	1.1	69	0.72
Restless legs	0.6	0.6	83	0.76
Changes to skin	0.6	0.5	75	0.58
Diarrhoea	0.3	0.2	78	0.42
Thirst	1.3	1.3	58	0.62
Anxiety	1	1	61	0.53
Family/friends’ anxiety	1.9	1.7	61	0.61
Depression	0.6	0.7	70	0.61
Felt at peace	1.4	1.5	60	0.51
Able to share with family/friends	1.4	1.4	53	0.43
Information	1.1	1.2	57	0.33
Practical problems	1	0.8	73	0.59
Time wasted on appointments	1	0.7	74	0.51

Interrater reliability for patients and staff was based on data from the first time point. For 11 items out of 24, we found agreement between the staff and patient assessment weighted kappa > 0.4, with the highest level of agreement for pain (0.66) and changes to the skin (0.56). The lowest level of agreement was found for anxiety (0.17). For the total score, the ICC was 0.73 (95% CI = 0.6–0.8) (see Table 3).

**Table 3:** Interrater reliability measured by weighted kappa and level of agreement (*N* = 88)

Item	Weighted kappa	% level of agreement
Pain	0.66	65
Shortness of breath	0.54	65
Weakness or lack of energy	0.36	43
Nausea	0.33	69
Vomiting	0.55	70
Poor appetite	0.36	68
Constipation	0.44	75
Sore or dry mouth	0.28	49
Drowsiness	0.41	47
Poor mobility	0.49	49
Itching	0.45	57
Difficulty sleeping	0.3	35
Restless legs	0.38	68
Changes to skin	0.56	70
Diarrhoea	0.43	81
Thirst	0.42	41
Anxiety	0.17	23
Family/friends’ anxiety	0.26	34
Depression	0.35	43
Felt at peace	0.39	49
Able to share with family/friends	0.3	35
Information	0.22	32
Practical problems	0.41	50
Time wasted on appointments	0.35	61

T1 = first time point, T2 = second time point after 3 days

### Sensitivity to change

Table 4 represents the change in IPOS scores between the first time point (T1, baseline) and the third time point (T3 after 1 month). Patients who reported positive changes after 1 month had a positive mean change in the total scores of eight points (a lower level of total score indicates less severe symptoms and concerns). Similarly, patients who reported negative changes showed a negative eight-point difference between the time points T1 and T3, signifying an increase in IPOS scores and more severe symptoms and concerns.

**Table 4:** Mean total IPOS-r score changes (between T1 and T3) by global change scale

	N (88)	Mean change T1–T3 (95% CI)
Yes, positive change	4	8.25 (4.08 to 11.9)
Yes, negative change	10	−8.6 (−11.1 to −4.9)
No change	47	0.6 (−2 to 2)
Missing data	27	

T1 = first time point, T3 = third time point, 1 month after the first time point

### Validity

Convergent validity was assessed using Spearman correlation with items from KDQOL-SF 1.2. Most of the correlations were in the range of 0.4–0.8 (Table 5). Only questions about family anxiety, practical problems, information needs, and time wasted on appointments did not have a significant correlation with items from the KDQOL-SF 1.2.

**Table 5:** Spearman correlations of IPOS-r and KDQOL items (*N* = 88)

Items from IPOS-r	Items from KDQOL Spearman correlations between IPOS-r and KDQOL-SF 1.2				
Pain	KDQOL7 0.77^b^	KDQOL8 0.69^b^			
Shortness of breath	KDQOL14f 0.76^b^				
Weakness or lack of energy	KDQOL9a 0.54^b^	KDQOL9e 0.56^b^	KDQOL9g −0.58^b^	KDQOL9i −0.68^b^	KDQOL14i 0.72^b^
Nausea	KDQOL14k 0.58 ^b^				
Vomiting	KDQOL14l 0.49^b^				
Poor appetite	KDQOL14h 0.69^b^				
Constipation	Not available				
Sore or dry mouth	Not available				
Drowsiness	KDQOL14i 0.55^b^				
Poor mobility	KDQOLsum 3a-3j − 0.68^b^				
Itching	KDQOL14d 0.8^b^				
Difficulty sleeping	KDQOL18a 0.68^b^	KDQOL18b −0.4^b^	KDQOL18c 0.28^a^		
Restless legs	KDQOL14j 0.33^b^				
Changes to skin	KDQOL14e 0.37^b^				
Diarrhoea	Not available				
Thirst	KDQOL15a 0.48^b^				
Anxiety	KDQOL9b −0.43^b^	KDQOL15f 0.45^b^			
Family/friends’ anxiety	KDQOL12d −0.32^b^				
Depression	KDQOL9c −0.55^b^	KDQOL9f −0.48^b^	KDQOL9h 0.49^b^		
Felt at peace	KDQOL9d 0.51^b^				
Able to share with family/friends	KDQOL19b −0.14				
Information	KDQOL23 −0.14	KDQOL24b 0.26^a^			
Practical problems	KDQOL24a −0.12	KDQOL15e 0.04			
Time wasted on appointments	KDQOL12b −0.08				

^a^significant at 0.05 level

^b^significant at 0.01 level

### Appropriateness and acceptability

The IPOS-r was feasible and acceptable for the patients and the staff. They appreciated its clarity and shortness. The average time to complete the measure was 9 minutes, which was acceptable to all participants.

## Discussion

Patient-reported outcome measures (PROMs) are very useful tools to capture patients’ experience with the disease and health care [7, 10]. Translation and validation of PROMs are needed, as they are used not only in clinical practice but also in research and auditing. The IPOS-renal measure does not have any validated translations except the English version.

The aim of this study was to adapt the IPOS-R to Czech. The Czech translation and cultural adaptation of IPOS-r were performed successfully, and no major changes were required after cognitive interviews except for adding a description of restless leg syndrome. The IPOS option “cannot answer”, which was suggested by participants of the cognitive interviews for psychological domains, was not used by our participants in this study; therefore, it was omitted. The Czech IPOS-r version is short, and the time needed to complete it is acceptable for patients and staff.

### Item analysis

Item analysis showed that all of the items in the IPOS-r met the requirements for item difficulty and item-total correlation. The lowest discriminant ability was found in the item vomiting because 91% of patients did not report this symptom. This is consistent with previous results and validation of the parent measure Czech IPOS on palliative patients [14]. Another study with patients from hospitals and home-based palliative services found similar results when vomiting, practical matters, and having enough information did not have a full range of responses [12].

### Reliability and internal consistency

The IPOS-r showed sufficient internal consistency, excellent test-retest reliability, and moderate agreement between the staff and patient assessment, especially in physical domains. In some of the physical domains, namely, weakness, nausea, poor appetite, difficulty sleeping, restless legs and sore or dry mouth, the study showed lower than moderate interrater agreement, so clinicians should focus on the assessment of these symptoms, as they seem to be overlooked.

Weighted kappa values for interrater reliability were sufficient for physical items (such as Pain or Changes to the skin), but they were in the range “poor” only for anxiety; lower than moderate agreement was seen in most nonphysical domains (feeling at peace, depression, ability to share feelings with family, time wasted on appointments), which are more difficult to assess. Similar results were also found in a study assessing the psychometric properties of the original English version of the IPOS-r [13, 25].

### Validity

Similarly, we were able to demonstrate good convergent validity for IPOS-r when compared to the KDQOL-SF 1.2 measure in most domains. This could signal redundancy of IPOS-r when compared to KDQOL-SF1.2, but the latter is not clinically used due to its length and extensiveness. An advantage of IPOS-r may be that the measure is able to cover similar domains to the KDQOL-SF 1.2 while at the same time being shorter and being more feasible for routine clinical measurement. Most of the correlations were in the range of 0.4–0.8, which indicates good convergent validity. The only items without sufficient correlation between IPOS-r and KDQOL-SF1.2 were family anxiety, ability to share with family, need for information, practical problems, and time wasted on appointments. These domains are not covered by the KDQOL exactly, so we matched them with similar concepts, which could have lowered the convergent validity. Diarrhoea, constipation and sore or dry mouth concepts were not present in KDQOL, so correlations were not assessed for these symptoms. As we do not have any other measure for quality of life available in Czech, we were not able to confirm the validity of the IPOS-r for all items, and this needs further investigation.

### Sensitivity to change

We tried to assess sensitivity to changes in IPOS-r. However, due to the small sample size, we were able to show only trends that need to be further investigated. Sensitivity to change of the original IPOS was also approved by other studies [12, 25]. Because of the small number of patients reporting the relevant change in 1 month, we could not calculate the Wilcoxon signed-rank test.

### Limitations

This study has several limitations.

Patients were asked to participate by the health care professionals who were involved in the patient’s care, which can be ethically problematic. We addressed this in the informed consent, where we explicitly stated that refusing participation would not have any adverse implications for the clinical care.

The numbers of patients in the study were not sufficient to provide factor analysis and assess the domains of the Czech IPOS-r. We determinated a sample size by exploring similar validation studies of IPOS translations and chose for the similar sample size as they did. In 12 out of 100 patients, data were not completed, which is common in end-of-life research, and were excluded from the analysis as in other validation studies to perform item analysis, which is plausible in this situation [26–28]. The renal patients in our study were very stable, with only 14 of 88 reporting a change in their health status after 1 month, so we could not calculate sensitivity to change by a statistical test. There were also some incomplete IPOS-r responses at time point three, 1 month after baseline, which could have been associated with lower compliance after a longer time period and deterioration of the patients’ health. The interval of the retest should be longer than 1 month to be able to assess sensitivity to change; on the other hand, this could increase the recall bias. The IPOS-r and KDQOL, which were used as gold standards [29], do not completely cover the same concepts; some domains assessed by IPOS-r are completely missing in KDQOL and vice versa, which can lower the convergent validity of the survey. However, the validity of the IPOS-r was also confirmed in cognitive interviews.

## Conclusions

The process of translation and cultural adaptation of the IPOS-r was successfully provided, and our study proved that the Czech IPOS-r is a responsive, reliable, and valid tool. There is no validated measure used by the Czech nephrologist in clinical care.

Our results recommend the use of the IPOS-r measure for the documentation of symptoms and concerns in patients with advanced chronic kidney disease, who are treated by either conservative management or dialysis.

## Supplementary Information

**Additional file 1: Supplementary Table.** Items from the IPOS-r matched to the items from KDQOL-SF covering the similar constructs and their estimated correlations.

## Data Availability

The datasets used and/or analysed during the current study are available from the corresponding author on reasonable request.

## Abbreviations

ESRD: End-stage renal disease
QoL: Quality of life
PROMS: Patient-reported outcome measures
IPOS-r: Integrated Palliative Outcome Measure-renal
KDQOL-SF: Kidney Disease Quality of Life Survey-short form
T1: First time point
T2: Second time point
T3: Third time point
ICC: Intraclass correlations
M: Mean
SD: Standard Deviation
POS: Palliative Care Outcome Scale
ISPOR: International Society for Pharmacoeconomics and Outcomes Research
