# Supplementary material for: Translation, cultural adaptation, and validation of the Integrated Palliative Outcome Scale-renal (IPOS-r) to Czech
Source: BMC Palliat Care. 2022 Aug 30;21:152. doi: 10.1186/s12904-022-01044-w (PMC9425933; doi:10.1186/s12904-022-01044-w)
Supplement: Supplementary file 1 — Additional file 1: Supplementary Table. Items from the IPOS-r matched to the items from KDQOL-SF covering the similar constructs and their estimated correlations. [file 12904_2022_1044_MOESM1_ESM.docx]

**Supplementary table: Items from the IPOS-r matched to the items from KDQOL-SF covering the similar constructs and their estimated correlations.**

| Items from IPOS-r | Items from KDQOL |  |  |  |  | Correlation |
| --- | --- | --- | --- | --- | --- | --- |
| Pain | KDQOL7 How much pain have you had in the past 4 weeks? | KDQOL8 During the past 4 weeks, how much did pain interfere with your  normal work (including both work outside the home and  housework)? |  |  |  | R > 0.70 |
| Shortness of breath | KDQOL14f  During the past 4 weeks, to what extent were you bothered by each  of the following? – shortness of breath |  |  |  |  | R > 0.70 |
| Weakness or lack of energy | KDQOL9a  These questions are about how you feel and how things have been with you during the past 4 weeks. How much of the time during the past 4 week did you have a lot of vigor? | KDQOL9e  These questions are about how you feel and how things have been with you during the past 4 weeks. How much of the time during the past 4 week did you have a lot of energy? | KDQOL9g  These questions are about how you feel and how things have been with you during the past 4 weeks. How much of the time during the past 4 week did you feel exhausted? | KDQOL9i  These questions are about how you feel and how things have been with you during the past 4 weeks. How much of the time during the past 4 week did you have feel tired? | KDQOL14i  During the past 4 weeks, to what extent were you bothered by each  of the following? Washed out or  drained? | R> 0.70 |
| Nausea | KDQOL14k During the past 4 weeks, to what extent were you bothered by each  of the following? Nausea or upset stomach |  |  |  |  | R> 0.70 |
| Vomitting | KDQOL14k During the past 4 weeks, to what extent were you bothered by each of the following? Nausea or upset stomach? |  |  |  |  | R> 0.70 |
| Poor appetite | KDQOL14h During the past 4 weeks, to what extent were you bothered by each of the following? Lack of appetite? |  |  |  |  | R> 0.70 |
| Constipation | Not available |  |  |  |  | Not available |
| Sore or dry mouth | Not available |  |  |  |  | Not available |
| Drowsiness | KDQOL14i During the past 4 weeks, to what extent were you bothered by each  of the following? Washed out or  drained? |  |  |  |  | R > 0.70 |
| Poor mobility | KDQOLsum 3a-3j  Some people are bothered by the effects of kidney disease on their  daily life, while others are not. How much does kidney disease  bother you in each of the following areas? |  |  |  |  | R > 0.70 |
| Itching | KDQOL14d During the past 4 weeks, to what extent were you bothered by each  of the following? Itching? |  |  |  |  | R > 0.70 |
| Difficulty sleeping | KDQOL18a  How often in the last 4 weeks did you wake up at night and couldn't get back to sleep? | KDQOL18b  How often in the last 4 weeks did you get enough sleep? | KDQOL18c  How often in the last 4 weeks did you have difficulty staying awake during the day? |  |  | R > 0.70 |
| Restless leg | KDQOL14j During the past 4 weeks, to what extent were you bothered by each  of the following? Loss of sensation in hands or feet? |  |  |  |  | R> 0.70 |
| Changes to skin | KDQOL14e During the past 4 weeks, to what extent were you bothered by each  of the following? Dry skin? |  |  |  |  | R> 0.70 |
| Diarrhea | Not available |  |  |  |  | Not available |
| Thirst | KDQOL15a  Some people are more bothered by the consequences of kidney disease in everyday life, and some are not at all. How much does kidney disease bother you in the following areas? Restriction of fluid intake? |  |  |  |  | R > 0.70 |
| Anxiety | KDQOL9b  How often in the past 4 weeks have you felt nervous? | KDQOL15f  Stress or anxiety associated with the illness |  |  |  | R= 0.5-0.7 |
| Family/friends anxiety | KDQOL12d  I feel like I'm burdening the family |  |  |  |  | R= 0.5-0.7 |
| Depression | KDQOL9c  How often in the last 4 weeks have you been so depressed that nothing could cheer you up? | KDQOL9f  How often in the last 4 weeks have you felt pessimism and sadness? | KDQOL9h  How often in the last 4 weeks have you felt happy? |  |  | R= 0.5-0.7 |
| Felt at peace | KDQOL9d  How often in the last 4 weeks have you felt peace and calm? |  |  |  |  | R= 0.5-0.7 |
| Able to share with family/friends | KDQOL19b  How satisfied are you with support from family and friends? |  |  |  |  | R= 0.5-0.7 |
| Information | KDQOL23  Consider the care you receive in connection with dialysis. Regarding your satisfaction, how would you rate the kindness and interest shown to you? | KDQOL24b  The dialysis staff is helping me cope with kidney disease. |  |  |  | R= 0.5-0.7 |
| Practical problems | KDQOL24a  The dialysis staff encourage me to be as self-sufficient as possible | KDQOL15e  How much does kidney disease bother you in the following areas? Dependence on doctors and other medical personnel? |  |  |  | R= 0.5-0.7 |
| Time wasted on appointments | KDQOL12b  Kidney disease is taking up too much of my time. |  |  |  |  | R= 0.5-0.7 |
